# The Unsolved Jigsaw Puzzle of the Immune Response in Chagas Disease

**DOI:** 10.3389/fimmu.2018.01929

**Published:** 2018-08-24

**Authors:** Gonzalo R. Acevedo, Magalí C. Girard, Karina A. Gómez

**Affiliations:** Laboratorio de Inmunología de las Infecciones por Tripanosomátidos, Instituto de Investigaciones en Ingeniería Genética y Biología Molecular (INGEBI), Consejo Nacional de Investigaciones Científicas y Técnicas (CONICET), Buenos Aires, Argentina

**Keywords:** *Trypanosoma cruzi*, humoral immunity, cellular immunity, innate immune response, adaptive immune response, immune evasion, Chagas disease, parasite

## Abstract

*Trypanosoma cruzi* interacts with the different arms of the innate and adaptive host's immune response in a very complex and flowery manner. The history of host-parasite co-evolution has provided this protozoan with means of resisting, escaping or subverting the mechanisms of immunity and establishing a chronic infection. Despite many decades of research on the subject, the infection remains incurable, and the factors that steer chronic Chagas disease from an asymptomatic state to clinical onset are still unclear. As the relationship between *T. cruzi* and the host immune system is intricate, so is the amount and diversity of scientific knowledge on the matter. Many of the mechanisms of immunity are fairly well understood, but unveiling the factors that lead each of these to success or failure, within the coordinated response as a whole, requires further research. The intention behind this Review is to compile the available information on the different aspects of the immune response, with an emphasis on those phenomena that have been studied and confirmed in the human host. For ease of comprehension, it has been subdivided in sections that cover the main humoral and cell-mediated components involved therein. However, we also intend to underline that these elements are not independent, but function intimately and concertedly. Here, we summarize years of investigation carried out to unravel the puzzling interplay between the host and the parasite.

## Introduction

More than a century after its discovery, Chagas disease continues to be one of the main public health problems in South and Central America, with approximate numbers of 6–7 million people infected in the world, 100 million at risk of infection, 56 thousand new cases per year and 12 thousand deaths per year (1, 2). It is the main parasitic disease in the Western Hemisphere, and its socioeconomic boundary is 7.5 times greater than that of malaria (3).

The World Health Organization (WHO) includes it in the list of the 20 Neglected Tropical Diseases. This implies that, along with other clinically diverse infections, Chagas disease is strongly associated with poverty, flourishing in disadvantaged environments, and persisting especially in tropical areas, where the diseases of this group tend to coexist. The environments to which vectors are best adapted and where they thrive are often linked to precarious housing conditions, and this constitutes an additional nexus between disease and poverty. Likewise, the WHO also points out that these diseases “have traditionally ranked low on national and international health agendas. They cause massive but hidden and silent suffering, and frequently kill, but not in numbers comparable to the deaths caused by HIV/AIDS, tuberculosis or malaria” (4).

It was not until recently that due attention began to be given to a relatively modern factor that affects the distribution of Chagas' disease and expands its borders (5). Despite originally being limited by the ecology of the vector, human migratory phenomena from rural areas to urban centers and from endemic to non-endemic countries have generated new challenges for the epidemiological control of the disease (1, 3). Economic stagnation and political repression have stimulated, since the last two decades of the 20th century, different migratory waves conferring the disease a character of globalized public health problem (5). It is estimated that the prevalence of infection among all Latin American migrants living in Europe is 4.2%, while approximately 300 thousand immigrants carry the infection in the USA (3, 6). However, and as aggravating factor, the underdiagnosis index is estimated to be around 95%, and health professionals in non-endemic countries lack the necessary expertise to provide adequate care to the sick (6, 7). Also in the context of these demographic movements, Chagas disease interacts reciprocally with socioeconomic factors, such as labor formality and healthcare access, to deepen the disadvantages of already disadvantaged social sectors (1).

The routes of transmission and the life cycle of *T. cruzi* are illustrated and explained in further detail in Supplementary Figures 1, 2. When vectorially acquired, Chagas disease has two major phases. The acute phase lasts approximately 2 months and typically presents a high number of parasites circulating in the blood. In most cases symptoms are subclinical. When clinically observable, they tend to be unspecific (with the exceptions of unilateral palpebral oedema, called Romaña sign, and skin lesion known as chagoma) and usually vanish on their own, within a few weeks or months. If untreated, patients usually enter in the second phase of the disease, the chronic phase, which begins asymptomatic, and may so remain for the rest of their life. However, up to 30-40% of these patients develop clinical manifestations, being cardiomyopathy, and megaviscera (enlargement of the esophagus or colon), the most prevailing (6). Congenital Chagas disease, due to mother-to-child transmission, renders nowadays approximately 1–5% of the infected cases. It evolves like the vector-borne infection, with the same risk of developing clinical manifestations of chronic Chagas disease later in life, unless treated (8). On the other hand, orally-transmitted Chagas disease, mainly reported in the Amazon region, is associated with unusually severe and early clinical symptoms, and high fatality rates due to high prevalence of cardiac pathology (9).

Even after years of profuse research aiming at unveiling the mechanisms involved in the pathogenesis of Chagas disease, the reason why some patients stay asymptomatic while others progress to symptomatic affliction remains obscure. Two hypotheses have been laid on the table: one of them proposes that tissue damage is a direct consequence of the presence of live parasites, inducing chronic inflammation, while the other settles down on a self-reactive response triggered by molecular mimicry between parasite and host proteins. Certainly, these mechanisms are not mutually exclusive (10) and they may both contribute to the clinical outcome of the infection.

Independently of the mechanisms involved in pathology, the main underlaying actor is the immune response orchestrated by the host organism, and its interaction with the parasite. In this context, it is important to keep in mind the broad spectrum of activation profiles found in Chagas disease patients, which can be attributed to multiple factors: the infective load, the route of infection, the genetic background of the parasite (which is linked to the presence or absence of virulence factors) and of the host, the influence of neuro-endocrine factors on the adaptive response, among others (11).

After infection, *T. cruzi* induces a strong innate and adaptive immune response in mammals that plays a major role during the acute and chronic phases of the disease. Nonetheless, this response is not effective enough to achieve complete clearance of the parasite. In order to survive within the mammal host, and as a consequence of an extensive history of co-evolution, *T. cruzi* has evolved several sophisticated mechanisms to evade the immune system action, while not critically affecting its host.

In this review, we revisit the results of research that shed light on the interplay between *T. cruzi* and the different components of the innate and adaptive immune response, with a special focus on the human infection scenario. We also discuss current knowledge on the mechanisms of immune evasion that enable the parasite to persist within its host, and the role of the immune response in protection and pathogenesis in the context of Chagas disease.

## Innate immunity

### The complement system in *T. cruzi* infection

The complement system comprises more than 40 plasma circulating proteins which opsonize pathogens, recruit phagocytes to the infection site and, in some cases, eliminate the pathogen in a direct fashion. It functions as a cascade of proteolytic events that amplifies the signal generated by the presence of a pathogen to favor its efficient clearance. The first activation step can occur by three different ways, known as the classical, alternative and lectin pathways, all of which converge in the formation of a C3 convertase complex, and the consequent separation of C3 in C3a and C3b. The effector mechanisms of the complement depend on this step: the complement-promoted phagocytosis is mediated by C3b receptors expressed by neutrophils and macrophages, and C3a is a pro-inflammatory agent. Furthermore, C3b is necessary for the generation of the C5 convertase, which gives rise to C5a (a strong pro-inflammatory signal), and for the formation of pores on the pathogen's membrane.

**Figure 1:**
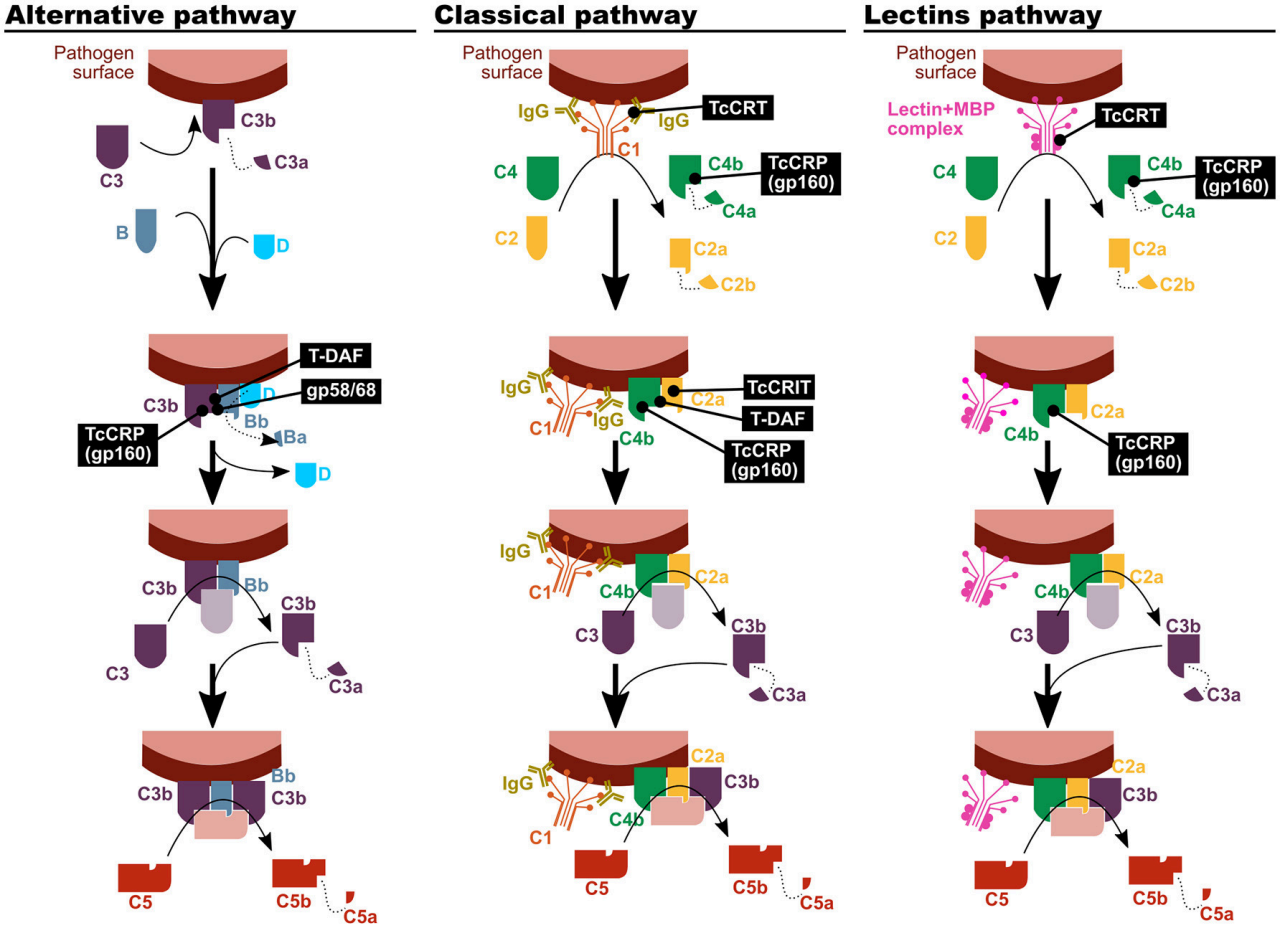
*T. cruzi* has mechanisms to inhibit the complement system. The parasite proteins involved in the modulation of the three complement activation pathways are indicated in black boxes. TcCRT, *T. cruzi* calreticulin; T-DAF, trypomastigote decay acceleration factor; TcCRP, *T. cruzi* complement regulatory protein; TcCRIT, *T. cruzi* complement C2 receptor inhibitor trispanning.

Although all *T. cruzi* forms are bound by complement factors, only epimastigotes, as well as metacyclic trypomastigotes of certain strains, are affected by their effector activities, mainly via the lectin and alternative pathways (12–14). Escape or subversion of the effector functions of the complement system is mediated by a set of molecules expressed on the parasite surface, which inhibit or dampen complement activation (Figure 1). Among them, calreticulin (TcCRT), trypomastigote decay acceleration factor (T-DAF), *T. cruzi* complement regulatory protein (TcCRP), *T. cruzi* complement C2 receptor inhibitor trispanning (TcCRIT), and gp58/68 have been studied the most (13, 14).

TcCRT is an endoplasmic reticulum (ER) protein that is translocated to the parasite surface after infection. It has been shown to be capable of binding different molecular pattern sensors, like C1q, mannose binding protein (MBP, also known as mannose binding lectin) (15) and L-ficolin (16). Therefore, it affects the first step on the classical and lectin complement activation pathways. Nevertheless, mannose binding lectin associated protein (MASP)-2 deficient mice suffer a compromise of the lectin activation pathway that is only partial, and as a result no differences with control mice were observed in susceptibility upon infection in this experimental model (17).

T-DAF is a protein from the inactive trans-sialidase family, and an analog of human DAF, which modulates the physiological decay of the alternative and classical pathway C3 convertase. In order to fulfill its function, both DAFs interfere in the C3bBb and C4b2a complexes formation, in the classical and alternative pathways respectively (13, 18, 19).

TcCRP, also known as gp160 (for 160 kDa glycoprotein) is another inactive trans-sialidase that is a glycosylphosphatidylinositol (GPI)-anchored to the cell membrane of the trypomastigote. TcCRP is capable of binding C3b and C4b, both in their soluble and bound forms, and it has been shown to interfere in the classical and alternative complement pathways (20). Furthermore, even though it has not been empirically tested, its C4 binding capacity could also affect the lectin pathway (13). Normally, it is not expressed on the epimastigote and amastigote forms surface, but their exogenous expression on epimastigotes renders them less susceptible to complement-mediated lysis (20, 21). Some authors have proposed a positive correlation between the TcCRP expression level and the virulence in different *T. cruzi* strains (22). However, evidence on that regards is scarce and inconclusive.

TcCRIT is a transmembrane protein homologous to *Schistosoma haematobium* ShCRIT (previously known as trispanning orphan receptor, TOR), and hCRIT in humans. These proteins have an extracellular acidic domain structurally similar to the C4 β chain, i.e., its C2 binding site. Hence, TcCRIT (like its analogs) and C4 compete for the binding of C2, therefore modulating the C3 convertase formation in the classical pathway (13, 14).

*Trypanosoma cruzi* 58/68 glycoprotein (gp58/68) functions as an analog of endogenous complement regulatory molecules, diminishing the alternative pathway C3 convertase formation by inhibiting the union of fB with parasite-bound C3b. Its release to the environment by trypomastigotes has also been reported, although the biological relevance of this mechanism is still to be clarified (13).

In addition to the direct interaction of these proteins with the complement factors, plasma membrane-derived vesicles [PMVs, (23)] released by infected host cells or by the parasite itself, inhibit complement functions (24). In the case of *T. cruzi* infection, molecules yet to be identified present in PMVs, impede C3b deposition on parasite surface, affecting the activity of C3 convertase (24). Even more, Wyllie and Ramirez demonstrated that PMVs isolated from a certain strain of the parasite can only confer resistance to complement-mediated lysis to parasites of their own same strain (25).

### Macrophages and neutrophils

Phagocytes, especially monocytes/macrophages, neutrophils and dendritic cells (DCs), are the first line of defense against pathogens on their way through the epithelial barriers. Monocytes and neutrophils express membrane receptors that recognize pathogen-associated molecular patterns (PAMPs) and damage-associated molecular patterns (DAMPs), enabling the detection and phagocytosis of microorganisms and cellular debris.

Among these receptors, some of the most relevant in the recognition of *T. cruzi* and its components are Toll-like receptors (TLRs). Up to now, a total of 10 TLRs have been identified in humans, and 13 in mice (26). TLR1,−2,−4,−5, and−6 are located on the cell surface, whereas TLR3,−7,−8, and−9 are expressed in intracellular compartments (27, 28). GPI-anchors derived from *T. cruzi* mucin-like glycoproteins (GPI-mucins) are ligands of TLR2 and−6, while glycoinositolphospholipids (GIPLs) are recognized by TLR4 (29–31). Furthermore, parasite DNA (which contains abundant oligodeoxynucleotide unmethylated CpG motifs) and total RNA have been identified as potent activators of TLR9 and−7, respectively (32, 33). In relation to this, not only monocyte subsets frequencies but also their expression of molecules related to PAMPs sensing and antigen presentation have been reported to be different between chronic Chagas disease patients and non-infected subjects. This appears to impact the profile of T_H_1, T_H_2, T_H_17, and T_reg_ cells (the role of which is discussed later), therefore highlighting the effect of phagocytes and antigen presentation on the establishment and profiling of long-term immune memory (34).

One of the principal means by which macrophages and other phagocytes contribute to the shaping of the response profile is the secretion of cytokines. In particular, the IL-12 family of heterodimeric cytokines is mainly produced by macrophages and DCs, and their signaling pathways polarize the immune response toward a broad diversity of pro- and anti-inflammatory profiles (35). With the aid of knock-out murine models, the role of the IL-12-related cytokines has been explored in experimental *T. cruzi* infection, consistently showing the importance of all the different components evaluated (36–40). However, the results of these studies are difficult to interpret, given that the knocked-out gene in each model is most often a component of more than one cytokine, and these in turn usually have opposite or pleiotropic effects. Table 1 summarizes the information obtained from these murine models, and data from experiments with patient samples that might be linked to observations in mice.

**Table 1:** Effects of the IL-12 family cytokines in *T. cruzi* infection.

**Knock out murine infection model**	**Affected cytokines/receptors**	**Observed effects**	**Possible correlate with human data**
**Mice:** IL-12p35^−/−^ (background: 129sv/Ev, backcrossed 5 times with C57BL/6) ***T. cruzi*****:** Tulahuen (37)	IL-12 (p35+p40) IL-35 (p35+Ebi3)	Reduced IFN-γ, TNF-α, and reactive nitrogen intermediate species at early infection, later normalized No NK and delayed T cell recruitment to the spleen Increased IL-18 production might partially compensate the lack of IL-12 by inducing IFN-γ secretion by spleen cells	IL-12 enhances *ex vivo, T. cruzi*-specific proliferative response from patients PBMC (41)
**Mice:** IL-12p40^−/−^ (background: C57BL/6) ***T. cruzi*****:** Y (38)	IL-12 (p35+p40) IL-23 (p19+p40)	Increased parasitaemia and mortality Impaired IFN-γ and NO production Higher titer of anti-*T. cruzi* IgG2b antibodies, similar titers of other isotype specific antibodies (vs. wild-type mice)	
**Mice:** Ebi3^−/−^(background: C57BL/6) ***T. cruzi*****:** Tulahuen (36)	IL-27 (p28+Ebi3) IL-35 (p35+Ebi3) IL-39 (p19+Ebi3)	Increased parasitaemia and mortality Increased expression of IL-4, IL-13, and IL-22 in spleen cells at early infection, later normalized Increased frequency of CD4^+^IFN-γ^+^and CD4^+^IL-17^+^ cells in spleen Increased IL-17A secretion by spleen cells upon *ex vivo* restimulation with anti-CD3 antibody at 7 dpi, normalized at 14 dpi Increased secretion of IL-4 upon *ex vivo* restimulation with anti-CD3 antibody. Improved survival rate upon *in vivo* anti-IL-4 treatment	
**Mice:** Ebi3^−/−^(background: C57BL/6) ***T. cruzi*****:** Y (42)		Increased mortality, but lower parasitaemia peak Increased expression of CCL5, CCR8, and CXCL10 coding mRNA in cardiac tissue, as well as of Th1 related genes Increased tissue parasitism in heart and liver Increased IFN-γ production in heart and spleen, and decreased production of IL-10 in heart Improved survival rate upon *in vivo* anti-IFN-γ or anti-NOS treatment	
**Mice:** WSX-1^−/−^(background: C57BL/6) ***T. cruzi*****:** Tulahuen (39)	IL27Rα (Receptor to IL-27)	Increased parasitaemia and mortality Augmented IL-4 production	Increased levels of IL-27p28 are related to milder clinical forms of Chagas disease (42)
**Mice:** IL-23p19^−/−^(background: C57BL/6) ***T. cruzi*****:** Tulahuen (40)	IL-23 (p19+p40) IL39 (p19+Ebi3)	Increased parasitaemia and mortality Impaired production of IL-17A	

The mechanism by which *T. cruzi* enters the so called professional phagocytes has been matter of controversy, since discordant results have been reported regarding whether it takes place by means of parasite-extrinsic mechanisms or the parasite participates actively in the process. On one hand, it has been shown that parasite internalization is inhibited by pharmacological blockade of actin polymerization in the phagocyte. On the other hand, it has been observed that even under such conditions the parasite can ingress to macrophages, supporting the active invasion hypothesis. Current consensus assumes that at least two different internalization mechanisms take place during infection: a phagocytic, actin-dependent one, and another one that relies on microdomain-like cell membrane structures on the macrophage (43, 44).

**Figure 2:**
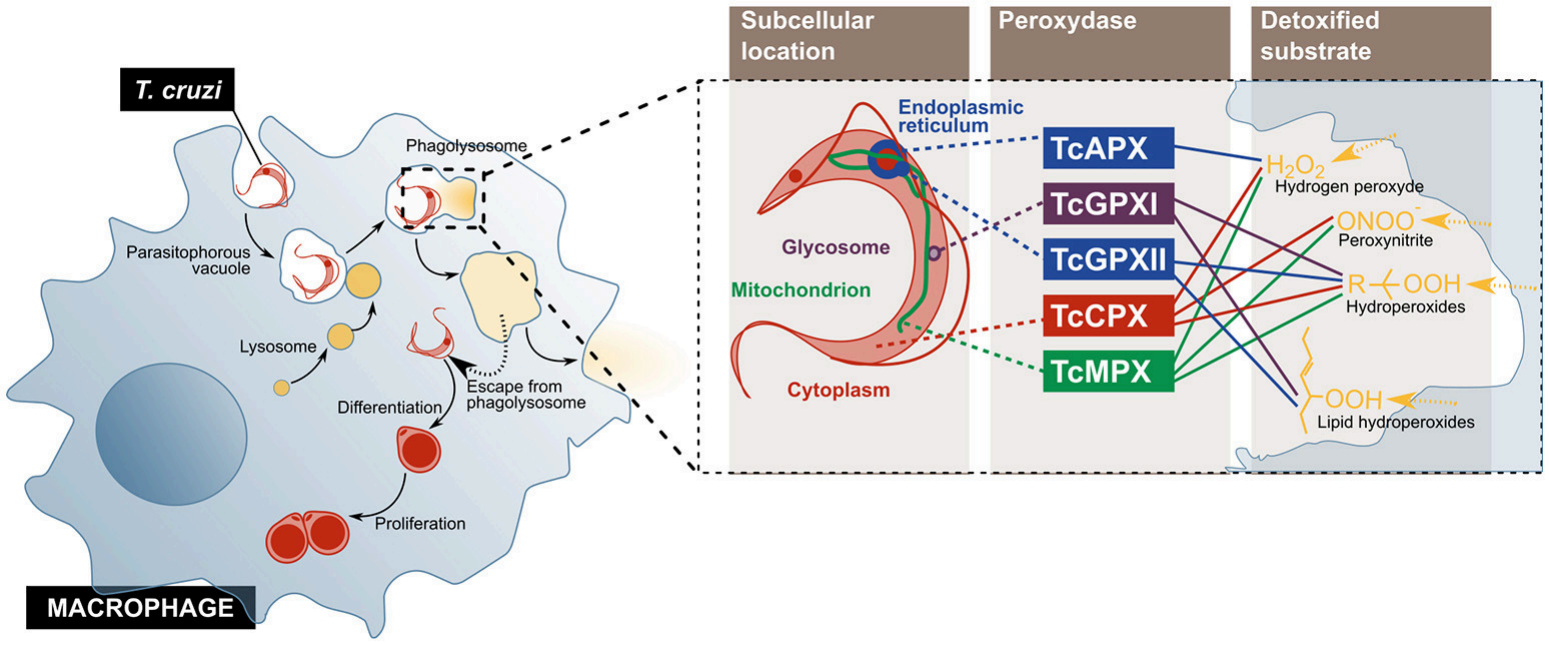
*T. cruzi* evades the microbicidal action of the macrophage by escaping from the phagolysosome into the cytoplasm, and by using enzymes that detoxify oxygen and nitrogen reactive species. The parasite enters the cell and escapes from the normal phagolysosome pathway, before its differentiation, and replication **(Left)**. On the **(Right)**, the peroxidases used by the parasite to inactivate different components of the oxidative burst imposed by activated macrophage are represented. TcCPX, *T. cruzi* cytosolic tryparedoxin peroxidases; TcMPX, *T. cruzi* mitochondrial tryparedoxin peroxidases; TcAPX, *T. cruzi* ascorbate-dependent haemoperoxidase; TcGPXI, *T. cruzi* glutathione peroxidases I; TcGPXII, *T. cruzi* glutathione peroxidases II.

Tissue resident macrophages are considered the first host cells to be invaded by *T. cruzi* upon infection. Although this cell type can internalize both trypomastigotes and epimastigotes, only the former can escape the phagolysosome (18, 44) (Figure 2). To detoxify the oxidant agents that the activated macrophage produces for its elimination, the parasite counts on an antioxidant metabolic network, composed of several enzymes and non-enzymatic molecules distributed across different sub-cellular compartments. Amongst them, five peroxidases have been described: the cytosolic and mitochondrial tryparedoxin peroxidases (TcCPX and TcMPX, respectively), which detoxify peroxynitrite, H_2_O_2_ and short chain organic hydroperoxides; the ascorbate-dependent haemoperoxidase (TcAPX), from the ER, that confers resistance to H_2_O_2_; and the glutathione peroxidases I and II (TcGPXI, located in the glycosome and the cytosol, and TcGPXII, in the ER), that enable the parasite's survival to lipid peroxides and hydroperoxides (18). Additionally, in the murine infection model, cruzipain, a cysteine-protease from the parasite, diminishes the macrophage's trypanocide activity by increasing the arginase activity, which competes with nitric oxide synthases, including iNOS, for their substrate, l-arginine (45).

It is worth mentioning that, despite an observed shrinkage of the morphologically macrophage-like population in flow cytometry experiments, the analysis of surface molecules expression demonstrated that monocytes with a pro-inflammatory phenotype have an increased frequency in pediatric Chagas disease patients, as compared with non-infected children of the same age (46).

The interaction between *T. cruzi* and neutrophils has been studied in murine model of susceptibility (BALB/c mice) and resistance (C57BL/6 mice) to infection with Tulahuen strain parasites (47). In the susceptible model, animals display increased parasitaemia, number of pseudocysts, and mortality when monocytes and neutrophils are depleted, as compared to non-depleted control mice. In contrast, the same depletion on the resistant model leads to decreased parasitaemia without changes neither in the number of pseudocysts nor in mortality. A comparative analysis of the secreted cytokines profile upon monocytes and neutrophils depletion suggested a protective role against infection for IL-2, IFN-γ, and TNF-α from macrophages, while IL-10 secretion seems not to be affected (47). Nonetheless, in a different study in which BALB/c mice were infected with Tulahuen strain parasites, neutrophils with a regulatory phenotype (IL-10 producers) were isolated from the spleen of *T. cruzi*-infected animals. In this model, cytokines signaled via heterodimers comprising the IL-17 receptor A (IL-17RA) produce opposite effects on parasitaemia upon neutrophil depletion: while wild type mice exhibit a decrease in this parameter, animals lacking this receptor component show increased number of blood-circulating parasites (48). Differences in the neutrophils induction of nitric oxide (NO) production on macrophages might explain the differential susceptibility to infection between BALB/c and C57BL/6 mice. This, in time, depends on TNF-α and elastase on one hand (a greater production of these molecules in C57BL/6 mice would have a protective effect), and prostaglandin E2 and TGF-β on the other (a greater production of these would lead to susceptibility in BALB/c mice, as a result of an increased parasite replication) (49). Nitrogen oxidant species have been demonstrated to be central to the control of infection in various murine models, mainly signaled through the IL-12/IFN-γ signaling axis (50, 51), but also involving TNF-α (51–53), IL-17A (40), and IL-18, the latter seemingly inducing IFN-γ secretion to compensate for the lack of IL-12 in a knock-out model (37).

In addition to phagocytosis and pathogen clearance by neutrophils in the context of the innate immune response, these cells can also produce fibrous structures made of DNA, histones, elastase and granular proteins, called neutrophil extracellular traps (NETs), by a special cell death program known as netosis. The NETs contribute to pathogen elimination, and their microbicide activity has been studied mainly in the neutralization of bacteria, but their role in the response toward other types of pathogens is not yet clearly understood (54, 55). Sousa-Rocha et al. demonstrated that trypomastigotes, and soluble *T. cruzi* antigens are capable of inducing NETs release on human neutrophils by activation of TLR2 and−4, and that this response depends on the activation of the respiratory burst and the production of reactive oxygen species. These NETs do not have trypanocide capacity, as they do not affect parasite viability, but they are able to reduce their infectivity by inducing trypomastigotes to differentiate into amastigotes in the extracellular environment (56).

### Dendritic cells

Like macrophages and neutrophils, DCs are also activated in the presence of PAMPs and DAMPs. *Trypanosoma cruzi* has been demonstrated to be internalized by DCs, although murine model experiments show there are different degrees of infectivity, associated to parasite strains. This dependence did not show any relation to Discrete Typing Units (DTUs) (57) nor to biological parameters of the DC (58).

**Figure 3:**
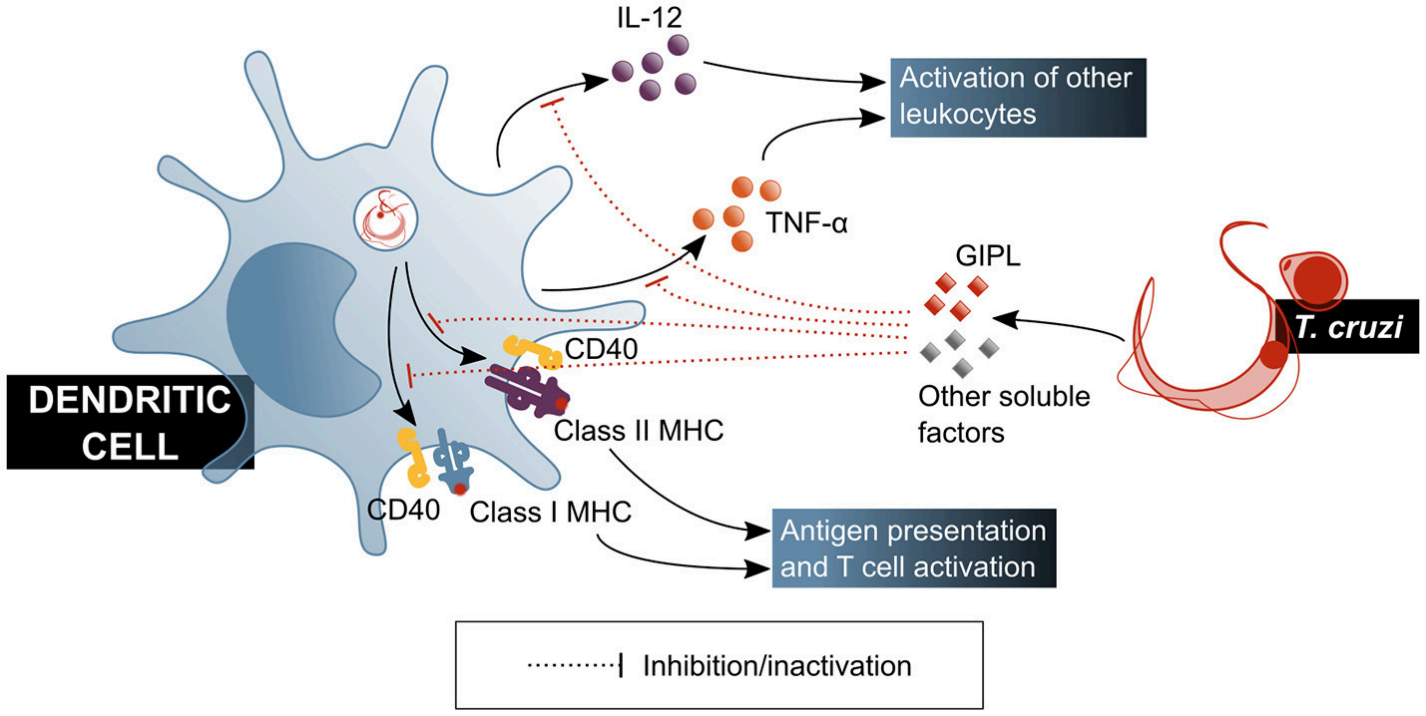
*T. cruzi* modulates the functionality of dendritic cells, affecting their ability to activate mechanisms of the adaptive immune response. GIPL, *T. cruzi* glycosylinositolphospholipids. Blue shaded boxes represent factors and mechanisms considered beneficial for the host.

Experiments with human DCs showed that their function is affected by factors secreted by the parasite, which induce a tolerogenic profile by decreasing IL-12 and TNF-α production (59) (Figure 3). It should be mentioned that under this model, cytoplasm invasion by the pathogen was observed. Also, a decrease of class I and II major histocompatibility complex (MHC) molecules and CD40 co-receptor expression was shown to be induced on DCs by *T. cruzi* soluble factors, tampering with this cell's antigen presentation capacity (59, 60). These effects have been attributed, at least partially, to parasite-produced GIPL (61), and have a direct consequence on T cell activation, as shown by a decrease in IFN-γ production by these cells in *in vitro* antigen presentation experiments, in which DCs were treated with the parasite prior to their usage as antigen presenting cells (APC) (60). Experiments with mouse bone marrow-derived DCs showed that inhibitory receptor SIGLEC-E is activated by the sialylated ligands on the parasite surface, downregulating IL-12 secretion and upregulating that of IL-10 (62), and may therefore be involved in immunomodulatory mechanisms associated to *T. cruzi* infection. This is in agreement with results by Ersching et al. who used a murine model to demonstrate that DCs that have been exposed to *T. cruzi* induce regulatory T (T_reg_) cells with an enhanced suppressor capacity on CD8^+^ T cells (63).

### Natural killer lymphocytes

**Figure 4:**
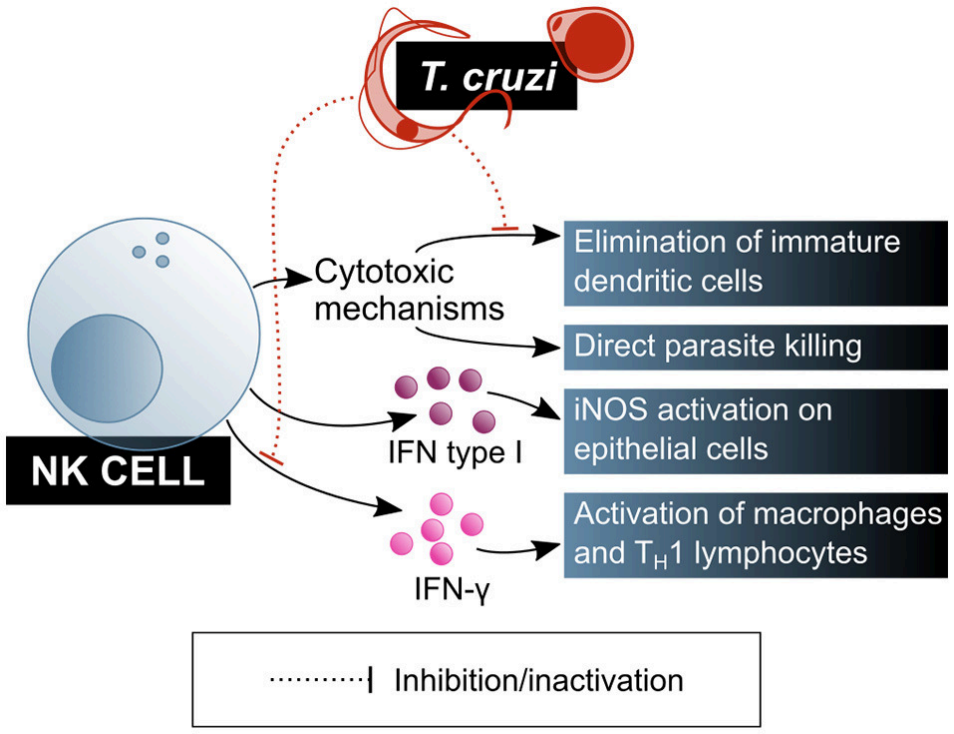
*T. cruzi* is a target of NK lymphocytes, which are one of the main cells of the innate immunity contributing to parasite clearance. However, *T. cruzi* also has mechanisms that modulate NK lymphocytes function. Blue shaded boxes represent factors and mechanisms considered beneficial for the host.

Natural killer (NK) cells play a central role in the innate immune response, especially in infections by intracellular pathogens. Cytotoxicity directed to the elimination of infected or damaged cells is one of their main effector mechanisms. Activated NK cells are also potent producers of IFN-γ, which activates macrophages and biases naïve T cell differentiation toward a T_H_1 profile. Both the cytotoxic and modulatory functions of NK cells are positively regulated by innate immunity cytokines, mainly IL-12, IL-15, IL-18, and type I interferons, which enhance cytotoxic capacity and IFN-γ production. As the role of these cells in *T. cruzi* infection is increasingly explored, their relevance has become more and more obvious (Figure 4).

NK cells' role on biasing T cell differentiation toward a T_H_1 profile and activating macrophages via IFN-γ secretion was demonstrated in the murine *T. cruzi* infection model. It has been shown that NK cells produce a peak of IFN-γ secretion shortly after infection, in a process that is dependent on adherent cells in the thymus but independent on T cells, and that also requires the presence of viable parasites (heat- or radiation-killed parasites failed to activate this cytokine secretion) (64). This IFN-γ blast may be crucial for the early control of parasitaemia during the acute phase of the infection. Depleting NK cells from these mice results in IFN-γ secretion abrogation and IL-10 production increase, probably causing the immune system to tolerate the presence of the parasite (64).

In addition, NK cells can eliminate extracellular parasites directly, by means of the quick formation of intercellular contacts that result in the immediate loss of motility and cell membrane damage for the parasite, as it was shown in mice. This mechanism depends on the NK cell activation by IL-12, and involves the exocytosis of cytotoxic granules, but is perforin-independent (65, 66), which led to the conclusion that the role of NK cells in controlling the parasite burden has more to do with direct extracellular parasite killing than with the elimination of *T. cruzi* infected cells.

The observation of a decrease in the infective capacity of the parasite on cultured mouse fibroblasts in the presence of total spleen cells, that is reverted when NK cells are depleted, has been pointed out as evidence for a regulatory role for this cell subset on non-immune cells in the context of *T. cruzi* infection. Looking into this phenomenon, Lieke et al. (67) corroborated that it is mediated by IFN-γ secretion from NK cells, which induces an increase in iNOS expression in the fibroblasts. Also, type I interferons were identified as messengers in this cross-talk between NK cells and fibroblasts, being produced by both cell types in response to the parasite. However, the effect of these interferons and IL-12 on the trypanocide mechanism induced by NK cells on fibroblast was demonstrated to be neglectable (67).

NK cells are also involved in the maturation of DCs: in normal physiological conditions, activated NK cells direct DC maturation by secreting cytokines while suppressing those DCs that do not undergo full, correct maturation. On that regards, murine model experiments have shown that live parasites, unlike their lysate, affect NK cells cytotoxicity on immature DCs, and that this effect causes greater parasitaemia and lower mice survival, without affecting parasite burden (66).

In the context of human infection, Ferreira et al. (68) conducted a microarray-based transcriptomic signature study on peripheral blood cells from Chagas disease patients, in which *ex vivo* transcriptional profiles were compared between patients classified in different categories: severe cardiopathy, mild cardiopathy, asymptomatic with negative PCR for *T. cruzi* DNA, asymptomatic with positive PCR, and control non-infected subjects. The expression of genes related to NK cells activity was found to be upregulated in positive PCR asymptomatic patients and mild cardiopathy patients, and downregulated in patients with severe cardiopathy (68).

A phenotypic study of the cell subsets circulating in peripheral blood from pediatric and adult Chagas disease patients, with and without cardiac symptoms, suggested a role for pre-NK cells in the activation of macrophage effector mechanisms, during early stages of the asymptomatic phase. These pre-NK cells are predominantly cytokine secretors, in contrast to mature NK cells which are predominantly cytotoxic. A greater frequency of mature NK cells was also observed in asymptomatic patients, as compared to cardiac patients, suggesting a contribution of this cell subset to the establishment and/or maintenance of the lack of symptoms during chronic infection (46). Additionally, peripheral and cord blood experimental infection assays highlighted NK cells as the most potent IFN-γ producing subset, besides also secreting IL-15 (69) in response to the pathogen, suggesting their relevance in the primary response to infection.

## Adaptive immunity

### B lymphocytes

B cells are known to play an important role in humoral adaptive immune responses by producing and secreting antibodies. However, they also have relevance in cytokine secretion and antigen presentation to other immune cells, therefore being a nexus between innate and adaptive immune responses. Moreover, in recent years, an increasing amount of studies identify B cell subsets with regulatory functions (70), suggesting that its purposes are much broader than previously thought.

**Figure 5:**
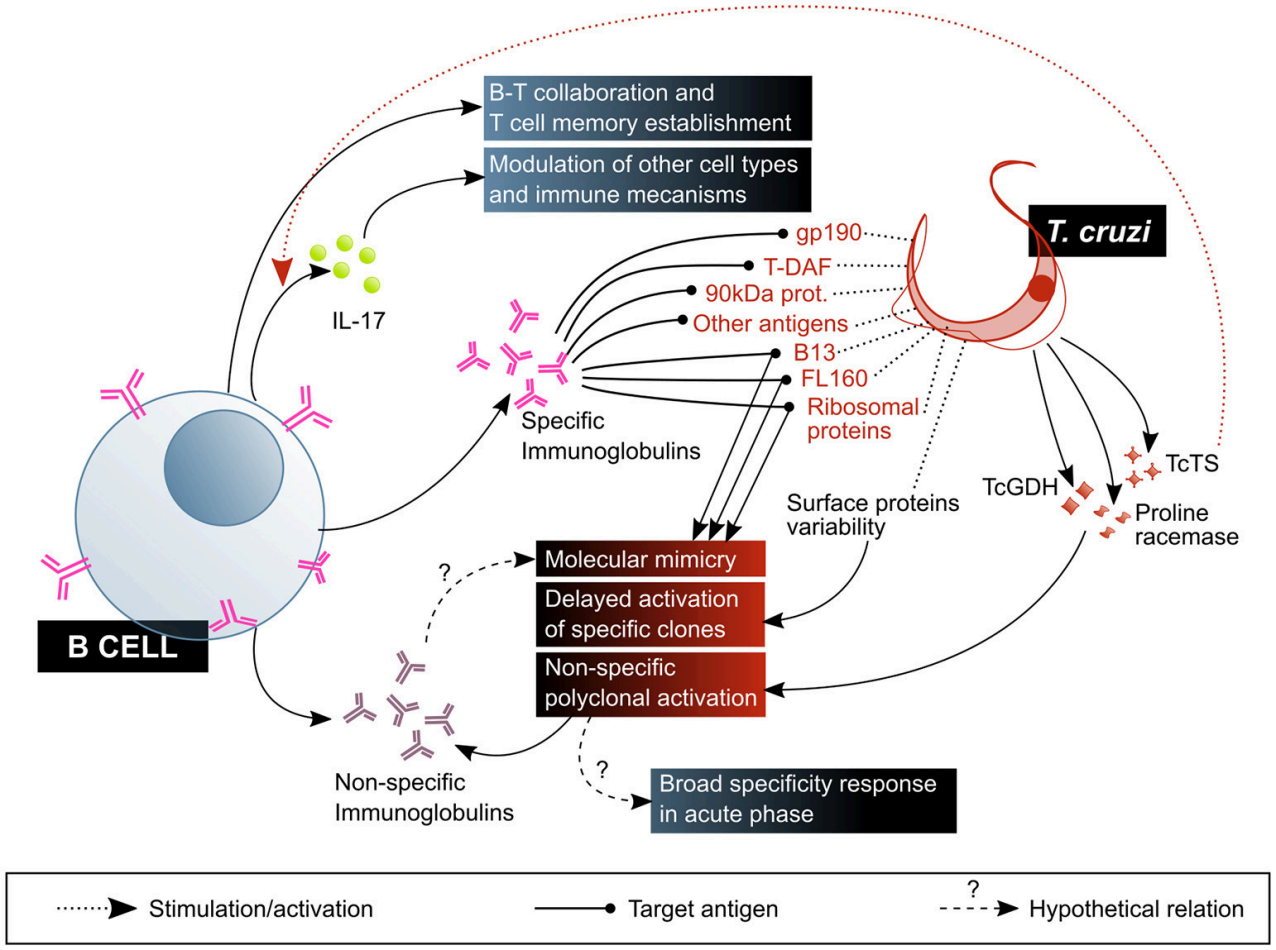
B cell-mediated response is central to the control of *T. cruzi* infection, but is also affected by parasite mechanisms that favor its escape and are associated with pathogenesis. The beneficial factors for the host are highlighted in blue shaded boxes, while the factors considered harmful to the host are shown in red shaded boxes. T-DAF, trypomastigote decay acceleration factor; TcTs, *T. cruzi* Transialidase; TcGDH, *T. cruzi* Glutamate dehydrogenase.

B cells and antibodies were amongst the first components of immunity to be studied in the context of Chagas disease, and as of today several aspects of their multiple roles remain obscure (Figure 5). The specificity of antibodies generated upon infection were the major focus of interest in the early days of this field. Up to now, the immune epitopes database (IEDB, http://www.iedb.org) has 89 *T. cruzi* molecule entries (plus several entries marked as “other *T. cruzi* protein”), which contain in total over 2 × 10^3^ epitopes bound by specific antibodies in human infection and in animal models. Some of these antibodies are often referred to as “lytic,” meaning they enable complement-mediated parasite lysis. Amongst target antigens to these antibodies are gp190, T-DAF, the 90 kDa surface protein and several GPI-anchored mucin-like glycoproteins. The mechanism by which these antibodies allow direct lysis of the parasites are thought to be related to the blockade of the complement evasion pathways discussed previously (71). Other proteins recognized by—non necessarily lytic—antibodies produced in response to *T. cruzi* infection are mucins (TcMUC), mucin-associated surface proteins (MASPs), trans-sialidases, amastigote surface proteins (ASPs), paraflagellar rod protein (TcPRP), kinetoplastid membrane protein 11 (KMP-11), glycoprotein gp82, the enzyme neuraminidase, heat shock protein hsp70, tubulin, ribosomal proteins, and several others (IEDB, accessed June 2018), besides carbohydrates like the so-called “gal epitope” (gal α1-3 gal) (72). It is worth mentioning that the recent application of high throughput technologies and informatics prediction methods have dramatically expanded the possibilities of exploring the repertoire of human antibodies produced against infection by this complex parasite, and constitute promising tools for the study of the humoral adaptive immune response in Chagas disease (73, 74).

The importance of anti-*T. cruzi* antibodies to the control of infection has been demonstrated in the mouse infection model: in spite of living significantly longer than T cell deficient animals, mutant mice incapable of producing antibodies (muMT^−/−^) are unable to control parasitic growth and succumb to infection during the acute phase (75).

Despite this, antibodies produced against *T. cruzi* do not effectively eliminate the parasite, providing an opportunity to establish a persistent infection. This deficient antibody response is possibly due to three main factors. The first is antigenic variability: the parasite exposes a variety of antigens on its surface, such as mucins, trans-sialidases and MASPs, encoded by highly polymorphic multigenic families. This high diversity of molecules expressed at the same time delays the activation of specific B cell clones, and therefore also the production and maturation of high-affinity antibodies with neutralizing capacity (18, 76, 77).

The second factor is the reduction of immature B cells in the bone marrow (BM), probably as a consequence of an increased apoptosis rate. It was proposed that, by affecting the BM, *T. cruzi* could compromise the whole humoral immune response, limiting the generation of mature B cells in the periphery (78, 79).

The third factor is non-specific polyclonal B cell activation: some parasite molecules have been demonstrated to cause non-specific, T-independent activation and proliferation of B cells in murine models of infection, generating splenomegaly and hypergammaglobulinemia associated to the production of non-*T. cruzi-*specific antibodies (18, 80–82). This phenomenon has been linked to susceptibility to infection in BALB/c mice in contrast with resistance in C57BL/6 mice. Monitoring total and anti-*T. cruzi* (measured as TcCRP-specific antibodies) IgM and IgG production, it was shown that while BALB/c mice do not produce IgM in response to the parasite, they suffer from hypergammaglobulinaemia and low parasite-specific response, and that C57BL/6 exhibit an initial increase in total IgG and IgM followed by a rise in anti-CRP antibody titer of both isotypes (83). A case of accidental infection permitted the observation of this phenomenon in the context of human acute infection, exhibiting expansion of total antibodies, initially IgM and IgA, followed by IgG with specificities not related to *T. cruzi* antigens (84). Glutamate dehydrogenase (TcGDH) (85), proline racemase (86), and TcTS (87) are amongst the parasitic proteins identified as polyclonal B cell mitogens. This polyclonal B cell activation may be functional to an early host defense with antibodies against a big spectrum of conserved structures present in *T. cruzi* and other pathogens, allowing the clearance of the parasite. Nonetheless, it might also be exploited by the parasite to avoid the host specific immune response: promoting a generalized expansion of the B cell subset, regardless of their specificity, would result in a diminished frequency of pathogen-specific antibody-producing B cells (79).

At this point, it is worth mentioning that one of the most widespread hypotheses explaining the pathogenesis of Chagas disease is the existence of self-reactive immune mechanisms developed as a consequence of infection (88). In particular, evidence of molecular mimicry has been found between host and parasite molecules, and it has been suggested that this may lead to the production of self-reactive antibodies with deleterious effects. Examples of this are *T. cruzi* peptide B13 and human myosin heavy chain, ribosomal P proteins and muscarinic and adrenergic receptors, and protein FL-160 and a neuronal 47 kDa protein, among others (89–92).

Given that self-reactivity has been observed most frequently in the chronic phase of Chagas disease in humans and experimental models, some authors propose that it is a result of a low level stimulation of self-reactive cells during a long period of time (93). However, cellular and humoral self-reactivity against myosin, and antibodies against actin and tubulin have been found in acutely infected mice (93, 94) and anti-laminin antibodies were detected in acutely infected humans (84), suggesting that this phenomenon may actually be initiated earlier, during the acute phase of infection.

B cell-deficient (muMT^−/−^ C57BL/6) mice have been used in experimental models of acute infection to test the relevance of these cells' contribution to anti-*T. cruzi* response. A deficient expansion of CD4^+^ and CD8^+^ T cells was observed in the absence of mature B cells, in particular within the memory T cell subsets, along with diminished T_H_1, T_H_17, and T_reg_ populations. This suggests that B cells are crucial for the establishment of *T. cruzi*-specific memory T cell populations which, as it is discussed later, are essential for controlling the infection. In the absence of mature B cells, the frequency of IFN-γ^+^CD4^+^ T cells decreases, while that of TNF^+^CD4^+^ T cells rises with concomitant higher plasma levels of this cytokine. In addition, the frequency of CD4^+^ T cells expressing inhibitory receptors is lower than in wild type infected mice, which may imply hampered regulation of these populations. All of these contribute to an overall exacerbated, unconventional and deleterious pro-inflammatory state (95, 96). Experiments in a murine model of vaccination with a TcTS peptide confirmed some of these observations, and added that even though B cells are indispensable for the systemic adaptive response generated by this vaccine, they would not be necessary for the vaccine-induced response in the mucosa (although experimentally observed in the spleen) (97).

An unexpected capacity of B cells, first observed in the murine model of infection with *T. cruzi*, is that of producing IL-17 to an extent even greater than T_H_17 cells (which are often considered as the main source of this cytokine) do (95, 98). Remarkably, this response seems to be activated by the enzymatic activity of the parasitic TcTS on the CD45 receptor expressed on the surface of the B cell, and depends on a signaling pathway and a transcriptional program different from those that direct T_H_17 cells activation (98). These authors also demonstrated that human tonsillar B cells, obtained from non-infected donors, secrete IL-17 when co-cultured with *T. cruzi* trypomastigotes. In this set-up, anti-TcTS antibodies blocked this activation pathway (98). This is in agreement with a previous observation by Magalhaes et al. who pointed out that a large proportion of the IL-17^+^ lymphocytes found in peripheral blood from Chagas disease patients are CD4^−^ and CD8^−^ (99). Altogether, these results highlight the role of B cells in the modulation and profile definition of the T cell response.

The frequency of blood-circulating B cells and particular B cell subsets is also linked to immunity and homeostasis, and is often affected by infectious processes. An increase in the frequency of circulating B cell seems to occur during the late acute phase of *T. cruzi* infection in humans, but only becoming significant at the beginning of the chronic phase (100). Nonetheless, it has been proposed that, in patients with established chronic Chagas disease, the number of CD19^+^ cells is not different from that of non-infected individuals (101, 102), suggesting a subsequent contraction of this population. Despite this apparent unaltered number of circulating B cells, the same is not true about the representation of the different B cell subsets: Fernández et al. reported a selective decrease in B cells from the memory subset (CD19^+^CD27^+^IgD^−^) with different degrees of differentiation (both IgM^+^ and IgG^+^), and in terminally differentiated plasma cells (CD19^+^CD27^+^CD138^+^), along with an increase in the unconventional double negative B cells (CD19^+^CD27^−^IgD^−^IgG^+^) (102). This implies, as the authors discuss, an increase in B cells incapable of generating a substantial anti-*T. cruzi* IgG antibody response. In addition, Fares et al. (101) found an augmented CD21-expressing B cell population, CD21 being a CD19-associated co-receptor which enhances the activation signals generated by membrane Ig in contact with complement-bound antigen and T cell-dependent response antigens (103).

Unlike the conventional (B2) cells, the B1 cell subset produces and secretes polyreactive natural antibodies and immunomodulatory molecules, which play an important role in protection against certain infections (104, 105). In mice, they constitute only a minor fraction of the B cells in spleen and lymph nodes, but they are the most abundant in the peritoneal and pleural environments (106). Infected BALB Xid mice carrying a mutation that prevents B1 cell development showed poor B cell response to *T. cruzi* infection and low levels of specific and non-specific antibodies in serum. However, they were able to control parasitaemia and developed almost no pathology in the early chronic phase of the disease (107). The resistance of these mice to experimental infection was attributed to the absence of IL-10 secreting B1 cells and high levels of IFN-γ (108). In this sense, Merino et al. (109), showed a decrease in peritoneal CD19^+^ cells in *T cruzi*-infected mice, with different kinetics for the B1 and B2 subsets. This was associated with an enhanced differentiation into plasma cells. Furthermore, atypical plasma cells called “Mott-like cells” were detected in the peritoneum of infected mice, containing high numbers of Ig^+^ cytoplasmic granules. Although the specific role of these cells in *T. cruzi* infection has not been clarified yet, the authors suggest that they might be associated with self-reactive responses observed in Chagas disease. Based on these results, it has been suggested that B1 cells would play a detrimental rather than protective role in Chagas disease (110). On the other hand, and as it was mentioned before, B1 cells may help to control the infection during the acute phase, by releasing high amounts of non-specific IgM and IgG, which rapidly decrease parasitaemia (109). Nevertheless, their precise role in the context of human infection is yet to be clarified.

Finally, regulatory B cells (B_reg_) secrete IL-10, IL-35, and TGF-β, and suppress autoimmune pathologies by hampering the expansion of pathogenic T cell clones and other pro-inflammatory lymphocytes, and promoting regulatory T cells (T_regs_) differentiation (70, 111, 112). Fares et al. described that patients with chronic Chagas disease have an increased frequency of IL-10 and TGF-β producing B cells in peripheral blood, both in basal state and upon *in vitro* stimulation with parasite lysate (101). It is yet to be determined whether, in the context of chronic Chagas disease, these cells have a benign effect by containing an overly inflammatory response, or detrimental by favoring tolerance toward the parasite presence.

### T lymphocytes

T cell response is initiated upon signals produced by the recognition of peptide-MHC complexes on the surface of APC by the T cell receptor (TCR). The ultimate consequence of this activation is the generation of a large number of effector, pathogen-specific T cells (T_E_), departing from a relatively small set of naïve T cells (T_N_) with diverse specificity. In the context of an acute infection, T cell response can be dissected in three phases: (i) priming and expansion, (ii) resolution and contraction, and (iii) memory (113).

During the first phase, T cells divide and differentiate into T_E_ cells, increasing their expression of CD69 (which results in augmented retention in lymphoid tissues), CD25 (IL-2Rα), CD40L (which enables induction of macrophages maturation and B cell helping functions), and CTLA-4 (an inhibitory receptor that modulates the response). Simultaneously, activated T cells increase other adhesion molecules and chemokine receptors that favor migration to and retention in lymphoid organs. Activated T cells may produce only one cytokine (monofunctional T cells), or simultaneously produce multiple cytokines (polyfunctional T cells). In general, polyfunctionality is considered a correlate of protection in the context of infection (114) and vaccination (115). Altogether, the changes that the T cell undergoes upon activation promote rapid amplification of specific response, generation of effector and memory populations, enhancement of APC functions, and circumvention of the response within non-pathological levels.

In the case of chronic Chagas disease, it has been shown that patients have an increased frequency of circulating activated T cells, and that these cells secrete pro- and anti-inflammatory cytokines (116). However, T cell proliferative response is hampered in Chagas disease patients, at least *in vitro*, upon exposure to a strong, non-specific mitogen, and decreased expression of receptors involved in T cell activation is observed. Furthermore, proliferation of T cells from non-infected donors is inhibited in the presence of *T. cruzi* antigens (117, 118). T cell response is particularly important for the maintenance of the typically low parasitaemia in the chronic phase of the disease (119), and if impaired (e.g., in VIH co-infection cases), it leads to rapid clinical onset (120, 121). However, exacerbated inflammation may lead to pathology and its regulation is key to controlling tissue damage and disease progression (6). T cells are central to this point since, as we discuss later, they have been linked to both the generation and the regulation of tissue inflammatory processes in the context of Chagas disease.

After the elimination of the antigen source, although this is not exactly the case in chronic infections, clonal contraction, takes place. The dominant process in this phase is death by apoptosis of the vast majority of the activated T_E_ cells, which allows the immune system to go back to its homeostatic state (122). Nevertheless, the activation of T_N_ cells gives rise not only to the great expansion of an effector population of selected specificity, but also to the differentiation of memory T cells (T_M_) that remain beyond the contraction phase. These have a remarkable capacity to self-renew by proliferation and perpetuate in the long term, and are specially efficient in acquiring effector functions rapidly upon a new event of activation (113). Within this population, two major subsets can be distinguished: central memory T cells (T_CM_) home at the lymph nodes, have a low effector capacity but high sensibility to stimulation and proliferation capability upon stimulation, specialize in long term immune protection; and effector memory T cells (T_EM_), homing at peripheral tissue, which can rapidly produce effector profile cytokines, but have a limited proliferative capacity (123, 124). On that regards, Fiuza et al. have reported that chronic Chagas patients have an increased frequency of circulating T_CM_ cells as compared to non-infected individuals, regardless of their clinical status (125), which might be attributed to a state of chronic activation.

In circumstances of persistent antigenic exposition, memory T cells may undergo a process termed exhaustion, characterized by the hierarchical loss of effector functions, altered expression and usage of transcription factors, metabolic disarrangements, and increased and sustained expression of inhibitory receptors (126, 127). Under normal physiological conditions, the latter take part in the control of inflammation and the contraction of T_E_ cell populations after antigen source elimination. These events are required for the uprising of functional T cell memory, and therefore, the conditions that lead to exhaustion result in the failure of a normal acquisition of a homeostatic memory T cell response (126).

In the murine chronic *T. cruzi* infection model, lack of cytokine production was observed in T cells recovered from tissue infiltrates, suggesting some degree of dysfunctionality (128). Despite some authors claim that the picture observed in this model does not fully fit the description of a T cell exhaustion process (121, 129), studies carried on chronic Chagas disease patients samples revealed the presence of exhausted T cells, exhibiting a direct relationship between their frequency and the severity of the cardiac disease (130, 131). Furthermore, an association between therapeutic success of benznidazole (one of the two anti-parasitic drugs of approved use for the treatment of acute Chagas disease) in pediatric patients, and the loss of parasite-specific IFN-γ producing T cells was observed (132). Also, the treatment of chronic patients seems to restore T cell populations functionality and composition, in terms of state of activation and differentiation (132, 133). This evidence supports the relevance of persistent antigenic stimulation during chronic *T. cruzi* infection in the development of an inefficient T cell response in the long term. In line with this, patients with discordant serological tests exhibit a broader, more diverse cellular activation profile against *T. cruzi* antigens than that of chronic Chagas disease patients with consistently positive serology. Hence, serodiscordant patients may be regarded as individuals who were once, transiently infected, but who achieved T cell-mediated clearance of the parasite, and as a consequence their humoral anti-*T. cruzi* response is circumscribed to basal, “resting memory” levels (134).

**Figure 6:**
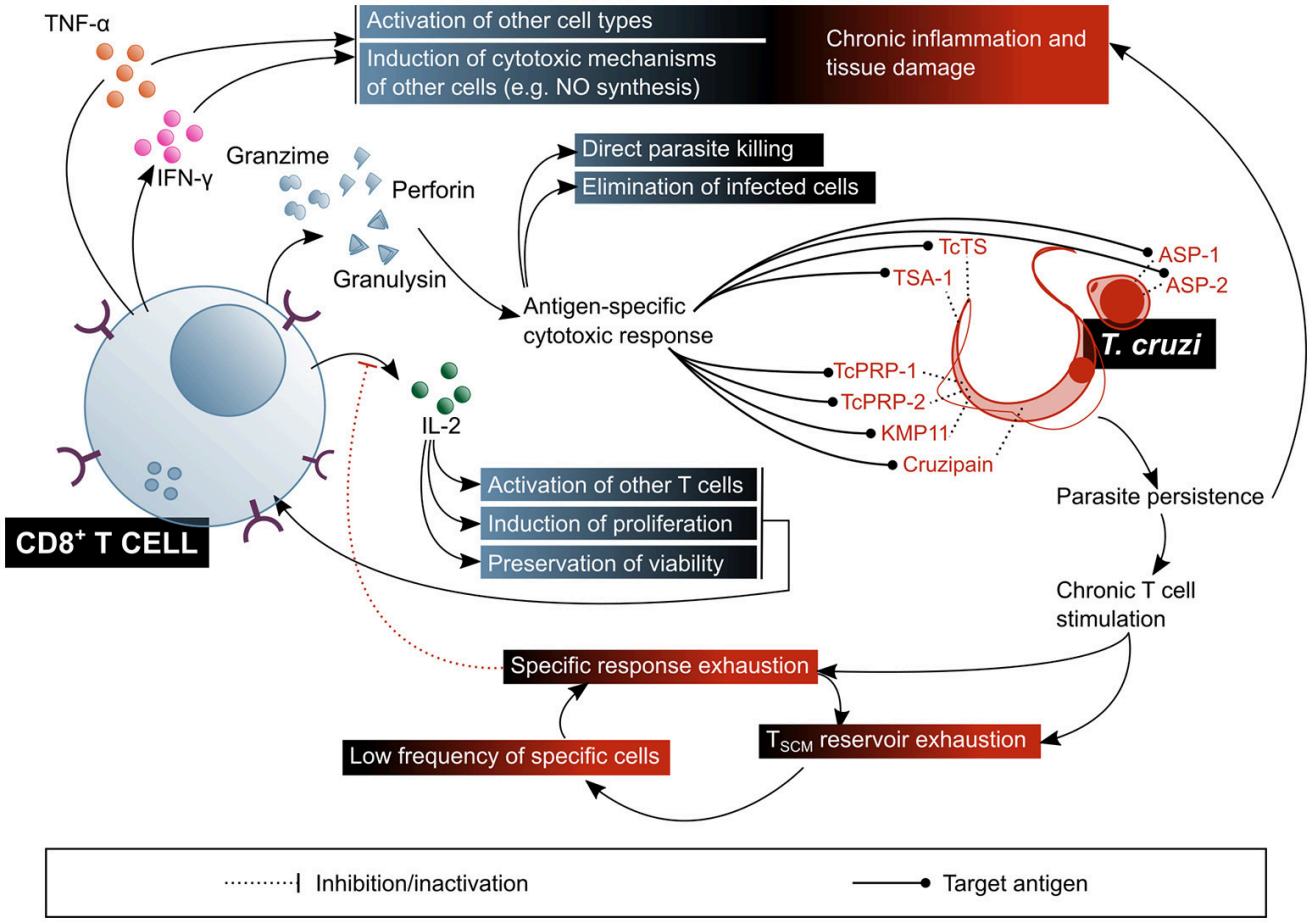
CD8^+^ T lymphocytes play a fundamental role in the control of parasitaemia and the elimination of cells infected with *T. cruzi*, but their chronic stimulation impairs their function. In addition, CD8^+^ T cells are involved in the pathogenic inflammatory processes of Chagas disease. The beneficial factors for the host are highlighted in blue shaded boxes, while the factors considered harmful to the host are shown in red shaded boxes. ASP, amastigote surface proteins; TcTs, *T. cruzi* transialidase; TcPRP, *T. cruzi* paraflagellar rod protein; KMP11, kinetoplastid membrane protein 11; TSA-1, trypomastigote surface antigen-1.

**Figure 7:**
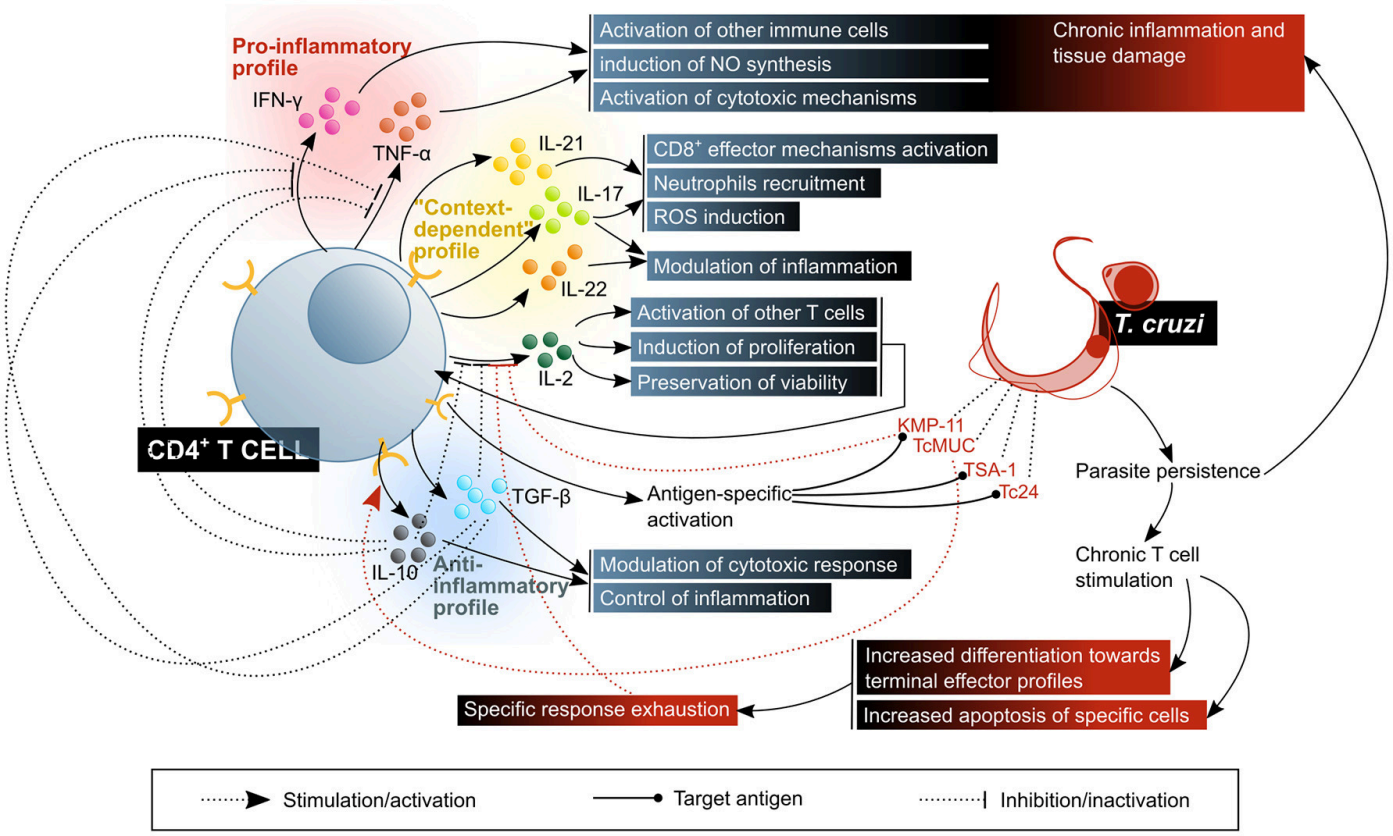
CD4^+^ T lymphocytes activate, enhance or modulate mechanisms of the immune response against *T. cruzi* infection. The control of the disease requires a balanced response in which there is a compromise between the activation of microbicidal functions and the control of inflammatory damage. The beneficial factors for the host are highlighted in blue shaded boxes, while the factors considered harmful to the host are shown in red shaded boxes. KMP11, kinetoplastid membrane protein 11; TcMUC, *T. cruzi* mucins.

Having established this general framework of available knowledge on T cell response to infection, and in order to facilitate their comprehension, the particulars of *T. cruzi* interactions with CD4^+^ and CD8^+^ T cells are approached separately, in the next subsections, and they are summarized in Figures 6, 7.

#### Cytotoxic T lymphocytes and elimination of the intracellular forms of *T. cruzi*

Activation of CD8^+^ T cells implies antigen recognition by the TCR in the context of a class I MHC molecule, and second signals provided by the APC, or by other lymphocytes, especially T_H_ cells. The latter are particularly relevant in situations in which antigen presentation is not very efficient and the innate response to the pathogen is relatively weak, which is the case of latent infections (135, 136). The result of this activation is the acquisition by the CD8^+^ T cell of the molecular machinery required for the elimination of the target cell, a process that constitutes the signature of CTL differentiation (137). Specifically, cytoplasmic granules containing proteins such as perforin and granzymes are developed, and these will later allow the CTL to eliminate other cells (138). Additionally, cytokine production, mainly IFN-γ, is triggered (135).

Given that the replicative phase of *T. cruzi*'s life cycle within the human host takes place in the intracellular environment, CTL response has been, within the last few decades, the predominant subject in the study of T cell response in the context of Chagas disease (Figure 6). It has been demonstrated that CD8^+^ T cells are an essential agent in the control of *T. cruzi* infection (29), as proven in murine models with depletion (139) or genetic deletion of this subset (140), in which animals do not survive the acute phase of the infection. It is also known that IFN-γ secretion is critical for the protective function of CD8^+^ T cells (29), and that one of the mechanisms by which this cytokine mediates protection is NO production (50, 53). Nonetheless, it would not be the only mechanism, since IFN-γ knock-out mice exhibit a different phenotype than iNOS knock-out mice, the former presenting a more severe onset and resembling that of CD4^+^ and CD8^+^ double-deficient mutants (141, 142). TNF-α has also been proven to be secreted by *T. cruzi*-specific CD8^+^ T cells isolated from Chagas disease patients (143). In addition, a reduction in the frequency of circulating CD8^+^ T cells in severe cardiac patients was observed in comparison with asymptomatic and mild cardiac patients, suggesting that CTL response might have a role in preventing the progression of the cardiac symptoms (144).

Despite the aforementioned, and given the experimental and clinical evidence, the deployment of a *T. cruzi* specific CD8^+^ T cell response does not imply protection against infection/reinfection. Diverse lines of research have tried to clarify the reason for which the established immune memory is uncapable of mounting an effective CTL response toward the parasite. One particular feature of this system, probably common to other parasites with an intracellular replicative phase, is that expansion and contraction of CD8^+^ T cells (experimentally assessed as cytotoxic activity and IFN-γ producing cells frequency of T cells against a TcTS and an ASP-2 epitopes in mice) occur with a certain delay in response to acute infection, as compared to the usual timings observed in responses toward viruses and intracellular bacteria (145). A likely explanation for this is that the initial infection goes unnoticed by the immune system, and therefore an effective activation of the innate response is withheld until the first round of replication and reinfection, which takes place 4–5 days afterwards (145–147).

Stem cell-like memory T cells (T_SCM_), a recently described subset, seem to be the primary reservoir of specific memory T cells, and to permanently replenish T_CM_ populations by proliferation and differentiation (124, 148–150). The persistent antigenic stimulus posed by chronic *T. cruzi* infection takes its toll on the maintenance of a functional memory population: it has been observed that patients with chronic Chagas disease have an imbalanced composition of the CD8^+^ memory T cell subset, with a lower frequency of T_SCM_ cells associated with a higher frequency of T_TE_ cells, as compared to non-infected subjects (151). Accordingly, a different study reports a higher frequency of CD8^+^CD45RA^+^IFN-γ^+^ T cells in cardiac Chagas disease patients, compared to that of asymptomatic Chagas patients and non-infected individuals (125). Although the authors of this article propose that this phenotype denotes that they are naïve CD8^+^ T cells, they might as well be terminally differentiated CD8^+^ T cells (152), which would further support the hypothesis of a progressive exhaustion of the CTL response associated to cardiac function compromise. In agreement with this, monofunctional CD8^+^ T cells have an increased frequency upon *in vitro* stimulation with parasite lysate in patients with severe cardiomyopathy, as compared to patients with milder forms of the disease (151). Additionally, a reduction of the T_EM_ subset in favor of an augmented terminal effector (T_TE_) subset within circulating CD8^+^ T cells has been demonstrated to occur in cardiac Chagas disease patients, which suggests that CTL-mediated protection against cardiac progression depends on the existence of an elevated number of competent CD8^+^ memory T cells, i.e., in non-terminal states of differentiation (144, 153). Also, an augmented expression of CTLA-4 on the surface of CD8^+^ T cells was observed in asymptomatic patients. Even though this was not the case for cardiac patients, an increment in the frequency of CD8^+^ T cells positive for intracellular CTLA-4 was detected (154). In partial discordance, an ulterior study showed a significantly increased frequency of CTLA-4 expressing CD8^+^ T cells in Chagas disease patients, with and without cardiac symptoms (155). Polymorphisms in the sequence of this receptor which affect its expression level may have an influence on the clinical outcome of the infection (156).

Another component of this dysfunctional response panorama is a deficient CD28-IL-2 signaling axis. Since IL-2 is required for the survival of an activated T cell and the generation and maintenance of T cell memory, a reduced secretion of this cytokine is likely to lead not only to a diminished effector response, but also to physical deletion of specific activated T cell clones (144, 157).

Regarding the specificity of CD8^+^ T cell response, several epitopes have been described, being those derived from proteins within the trans-sialidase proteins family the most thoroughly studied. In particular, TSKb20, a peptide predicted to bind mouse MHC class I molecule H-2K^b^, has demonstrated a high level of immunodominance in the murine infection model, with specific cells reaching up to 30% of the circulating CD8^+^ T lymphocytes (158, 159). This CD8^+^ T cell response, highly focused in a few epitopes, was proposed as a mechanism for the parasite to evade the immune response (159). However, it was later demonstrated that inducing tolerance toward the TSKb20 epitope has a negative effect on the control of parasitaemia in the murine model, which rules out the hypothesis of immunodominance contributing to parasite escape by impairment of a broader response (146). Furthermore, even though the observation of TcTS immunodominant epitopes is often extrapolated to *T. cruzi* infection in general, there is not sufficient evidence of a correlate in the context of human infection. A predictive approach similar to the one which led to the TSKb20 epitope was applied to the HLA-A2 supertype of human class I MHC alleles, pointing out similar trans-sialidase epitopes, which induced diverse levels of reactivity in IFN-γ ELISPOT experiments with HLA-A^*^02:01^+^ chronic Chagas patients samples (158).

The family of trans-sialidases and related proteins has caught the interest of many researchers from the field of T cell immunology of Chagas disease. In addition to their aforementioned role in escape and immunomodulation, and the existence of T cell epitopes derived from these proteins, they constitute an extremely diverse set of molecules, with about 1,400 different proteins annotated on the genome of *T. cruzi* (CL Brener strain), and an estimate of 1,800 unannotated variants. Conversely, *Leishmania* species lack this family of proteins, and in *T. brucei* it only has 6 members, which suggests this group of proteins was extraordinarily expanded when *T. cruzi* diverged from the other trypanosomatids (146). From an evolutive point of view, it is tempting to speculate on the relevance of this phenomenon for the success of *T. cruzi* as a parasitic organism, and it might therefore have a relevance for the immune response toward infection.

Other CD8^+^ T cell epitopes from *T. cruzi* trans-sialidase family proteins, confirmed as immunogens in HLA-A^*^02:01^+^ patients, are the trypomastigote surface antigen (TSA)-1-derived peptide 77.2 (160), amastigote surface proteins (ASP)-1 and−2 (161), paraflagellar rod proteins (TcPRP)-2 and−3 (162), cruzipain, and FL-160 (163). Kinetoplastid membrane protein (KMP)-11, is a non-trans-sialidase CD8^+^ T cell epitopes-containing protein, demonstrated to be recognized by cells from chronic Chagas patients. Other proteins that activate murine CD8^+^ T cells, but without confirmed immunogenicity in humans, are LYT-1, β-adaptin, paraflagellar Ca^+2^ binding protein (CaBP) and heat shock protein (HSP) (141, 164).

It is worth mentioning that, regardless of their specificity, an expansion of TCR-Vβ 3.1-expressing T cells was observed within the CD8^+^CD28^+^ subset in Chagas disease patients (165), suggesting a preferential activation of T cells expressing this TCR family in the context of *T. cruzi* chronic infection.

Several pieces of research have aimed at determining the frequency and phenotype of *T. cruzi*-specific CD8^+^ T cells utilizing parasite epitopes (TSA-1, ASP-1 and−2, CaBP, LYT-1) in ELISA-, ELISPOT- and intracellular cytokine staining (ICS)-based approaches. The success of these strategies has been strongly hampered by the low frequency of such cells in the circulation of chronic Chagas patients (130, 141, 158). This low frequency was shown to be exclusive for the *T. cruzi*-specific cells, by comparison with the response toward an influenza virus peptide in samples from the same subjects. Consistent evidence had already been obtained in the murine model, infecting mice with transgenic parasites expressing the heterologous antigen OVA, in secretory or GPI-anchored versions. Results indicated an initial expansion of OVA-specific CD8^+^ T cells, but these were only maintained in low frequencies (166). Different explanations were proposed for this feature. One of them involves the diversity of peptides incorporated to the class I MHC antigen processing and presenting machinery: if too many different epitopes are shown on the surface of an APC, it is possible that they will compete for antigen presentation and that in consequence none will reach a density higher than the threshold necessary for T cell activation. Another hypothesis relates it to the loss of effector function and peripheral tissue suppression (130, 141).

Besides its participation, with the mentioned nuances, in the elimination of the parasite during infection, a remarkable amount of evidence suggests that CD8^+^ T cells are also involved in tissue damage and inflammatory processes linked to the clinical manifestations of Chagas disease (167–169). Inflammatory infiltrates in patients with cardiac or digestive forms of the disease are rich in activated CD8^+^ T cells, and these express cytolytic molecules like granzymes and TIA-1 (116, 170, 171). A predominance of pro-inflammatory cytokines-producing cells in cardiac patients was also reported (172), although cytokine profile comparative studies between asymptomatic and cardiac Chagas disease patients showed discordant results: some authors state that IFN-γ secretion has a protective effect regarding the development of the symptoms (121, 130, 173), while others put forwards a harmful effect for this pro-inflammatory cytokine on cardiac function (173–175). A relationship between the T_TE_ enriched CD8^+^ T cells profile in cardiac patients and the cardiac damage itself has been proposed, given the enhanced cytotoxic capacity of this subset (151). The accumulated evidence on that regards highlights the importance of the regulation of the response in order to achieve a balance between an efficient effector activity and controlling inflammatory damage.

Interestingly, a recent study demonstrates that CTL have a direct trypanocide action on intracellular and circulating parasites (176). Results showed that, although it is not necessary for the elimination of infected host cells via perforin and granzyme B, granulysin is required for the intracellular parasites viability to be affected. Authors propose that granzymes enter the infected cells through the action of perforin, and from thereon penetrate amastigotes by means of granulysin. In regard to the circulating trypomastigotes, it has been proven that they can only be affected by granzyme B in the presence of granulysin. Notably, rodents lack this protein, which puts in evidence certain limitations of the classical murine model of infection for the study of CTL response in Chagas disease. This study also demonstrated that genetically engineered mice which express the enzyme have a better control of the infection and of the cardiac tissue damage, resulting in better survival rates (176).

#### Helper T cells and specific immune response modulation

CD4^+^ T cells are characterized by the expression of surface molecules and the secretion of cytokines which modulate the activity of other cells, mainly macrophages, DCs and other lymphocytes. Upon activation, they stimulate, via CD40L, DCs that had encountered pathogens, promoting their maturation and the production of polarizing chemokines and cytokines. This is critical for the induction of an appropriate CTL response. Some activated CD4^+^ T cells increase CXCR5 expression, which directs their migration toward the B cell follicles in lymphoid organs, where they interact with B cells and differentiate into follicular helper T cells (T_FH_), specialized in favoring the selection of specific B cells and inducing them to produce high affinity antibody production. The vast majority of proliferating activated CD4^+^ T cells differentiate into one of multiple T_H_ profiles, acquiring different migratory behavior and effector properties, and they enter circulation to extravasate into inflamed peripheral tissue. Once there and upon antigen recognition, they produce cytokines according to their T_H_ profile, in order to potentiate the activity of other immune system cells present *in situ*. The diversity of T_H_ phenotypes generated during primary response to an infection is important, since it will be mirrored by the diversity of memory T cells that will persist beyond infection (177).

It is known that in the chronic *T. cruzi* infection murine model, CD4^+^ T cells are a main component of the cardiac lesions infiltrates, which might suggest they are relevant to the response against the parasite (178). Nonetheless, and possibly due to the indirect nature of their physiologic role, i.e., their ultimate effect requires collaboration with other cells, little is known about their involvement in Chagas disease, especially in comparison with the extension to which CD8^+^ T cell response has been studied (Figure 7). A class II MHC knock-out mouse model applied to the study of the CD4^+^/CD8^+^ T cell collaboration led to the conclusion that CD4^+^ T cells are not absolutely necessary for the establishment of a CTL response (179). However, the specificity profile and migratory patterns of the expanded CD8^+^ T cells were not the same as those in wild type control mice, and transgenic animals presented a substantially higher susceptibility to infection, perishing to it in relatively short timespans (179). This not only limits the utility of this model for the evaluation of the role T_H_ cells play in the chronic *T. cruzi* infection scenario, but also demonstrates that, despite not essential for the generation and expansion of parasite-specific CD8^+^ T cells, CD4^+^ are indeed important for the control of infection and/or inflammation.

The variety of different T_H_ cell profiles, defined according to the cytokines they produce and the expression of typical transcription factors, is tightly linked to their function, since the predominance of one or other profile in the context of a response depends on the nature of the pathogen, and therefore enables the activation of the mechanisms most convenient for its elimination (177, 180). Results from the murine model suggest that, with regards to the level of protection that one or the other profile against *T. cruzi* infection, a coordinated response between T_H_1 and T_H_2 profiles is desirable (181, 182), with predominance, according to several authors, of T_H_1 effector mechanisms for the control/elimination of the parasite (183–185).

The mouse infection model has allowed the discovery of some parasite enzymes that affect the physiology of CD4^+^ T cells in diverse ways, constituting potential evasion mechanisms: two enzymes from the trans-sialidase family (a proper trans-sialidase and another one lacking this activity), inhibit their expansion and promote a T_H_2 cytokine profile, as authors observing a decrease in the secretion of IFN-γ and an increase in that of IL-4 suggest. Accordingly, a decreased expression of IL-2 and its receptor IL-2Rα (CD25), along with a reduction in TCR signaling, were observed, and IL-10 was demonstrated to be required for this to occur. Since the trans-sialidase activity seems not to contribute to this modulation, the authors propose that a lectin-surface glycoprotein binding-like mechanism is the cause of these effects (186). Similarly, TcMuc, a sialoglycoprotein on the surface of *T. cruzi*, was shown to produce similar effects on the expression of IL-2 and CD25, and therefore on the proliferation of murine CD4^+^ T cells, although cytokine secretion was more broadly affected in this case, involving not only IFN-γ, but IL-4, IL-10, and TGF-β as well (187). Here, the operating mechanism seems to be SIGLEC-E binding (as it was described for DCs), which is supported by the decrease in these effects when TcMuc is artificially desialylated (187). Another molecule modulating the function of these T cells is KMP-11, which diminishes IFN-γ secretion upon activation, without modifying that of IL-4 (188). All four agents mentioned, the active and inactive trans-sialidases, TcMuc and KMP-11, affect the whole population of CD4^+^ T cells, regardless of their specificity (186–188).

In the human infection, Albareda et al. (189) demonstrated an association of a lower frequency of *T. cruzi*-specific IFN-γ producing CD4^+^ T cells with the degree of severity in patients with Chagas chronic cardiopathy. The results on this report also suggest that most parasite-specific circulating CD4^+^ T cells in patients are newly-recruited cells, and they have a highly differentiated profile. Furthermore, in the most severe cases of cardiac disease, they present markers of apoptosis, while a minor fraction shows a long-term memory T cells phenotype (189). This evidence favors the T cell response exhaustion hypothesis in the context of chronic Chagas disease. In addition, experiments in which PBMC from chronic cardiac Chagas patients were stimulated *in vitro* and secreted cytokines were analyzed showed that antigens from the parasite induce a response profile that does not completely fit in any of the mentioned, including T_H_1 (IFN-γ, TNF-α) and T_H_2 (IL-4, IL-13) cytokines, along with IL-2, IL-10 and GM-CSF. GM-CSF, IL-10, and TNF-α secretion was attributed at least partially to the response against parasite ribosomal P proteins which, as mentioned before, are related to humoral self-reactivity phenomena (190).

Another subset of T_H_ cells, T_H_17, contributes to response against pathogens. Although early research on the subject emphasized their pathogenic role, especially in autoimmune disease or chronic inflammation models, evidence in favor of the protective role of these cells against infection by diverse pathogens has accumulated as well (191). They produce cytokines IL-17A, IL-17F, IL-22 and IL-26, and chemokine CCL20. A major proportion of these cells, often termed as a different T_H_17/T_H_1 subset, produce IFN-γ along with IL-17A. Their physiological relevance is still subject of controversy, since the effects of their activity differ depending on the contextual pathology and the experimental model of study (180). The main function of T_H_17 cells are the recruitment and activation of granulocytes/neutrophils by IL-17A-mediated activation of colony stimulating factors, and CXCL8 on macrophages and tissue resident cells. Meanwhile, IL-21 and IL-22 participate on mononuclear and tissue resident cells activation, and might therefore be involved in inducing or maintaining a chronic inflammatory process (177, 180, 191).

It has been demonstrated that T_H_17 cells can exert a potent protective function against *T. cruzi* infection in mice, by activating oxidative species production in infected cells via IL-17A, and CTL cytotoxic mechanisms via IL-21. Adoptive transfer of T_H_1 or T_H_17 cells specific against p7, a trans-sialidase epitope that results immunodominant in this model, was used to show that the latter confer greater protection against the parasite than the former (192). Although T_H_17 cells are markedly regulated by IL-12 family cytokines in the murine infection (193) and CD4^+^ T cells of this subset are a part of the inflammatory infiltrates in such model (194), the functional importance of this subpopulation has not been clarified yet. Nonetheless, it is clear that IL-17, the hallmark cytokine of this profile, is involved in the modulation and orchestration of the response against *T. cruzi*. IL-17A and IL-17F are produced in mice upon infection (48, 195), and deletion or depletion of these cytokines or their receptor consistently leads, in different models, to reduced survival and augmented tissue damage, accompanied by an enhanced pro-inflammatory response (40, 42, 195–197). In some of these IL-17 deficient models, decreased parasitaemia and/or tissue parasitism is observed as well (195, 42), which suggests that this cytokine might be playing a regulatory role, by limiting pro-inflammatory mechanisms, but at the expense of also restraining parasiticide effector functions. In some other models, a higher number of parasites is found in blood (197), indicating a role for this cytokine in controlling the infection. In addition, IL-22, a pleiotropic cytokine mainly produced by T_H_17 cells, is secreted in response to *T. cruzi* infection in mice, in an IL-23-dependent fashion, which seems to contribute to the regulation of inflammatory cytokines (198). With regards to human patients, the panorama is less clear: while some authors have shown that a higher frequency of IL-17^+^ cells within the CD3^+^CD4^+^ subset of PBMC is associated with milder forms of the disease (99, 199), and a correlation of IL-17A plasma levels with better cardiac function (200), others found increased levels of IL-17 in the sera of patients with higher risk of sudden death (201). However, it should be kept in mind that, as it was discussed above, T_H_17 are not the only cell lineage producing IL-17 in response to *T. cruzi*.

Advancements in the technologies applied to the study of the T cell biology, and knowledge generated in the last years of this field have enabled the distinction of other functional subsets of T_H_ cells, such as T_H_9 and T_H_22. However, no published data is available yet regarding their role and importance in the context of *T. cruzi* infection.

Regulatory T cells (T_reg_) operate tolerance mechanisms via an *in trans* action upon other T cells, thereby restricting potentially pathogenic immune responses (202). Under normal physiological conditions, they are generated mainly by recognition of self-antigens in the thymus, and of foreign antigens in peripheral lymphoid organs. Their regulatory function is mediated by IL-10 and TGF-β, the inhibitory receptor CTLA-4 and exoenzymes CD39 and CD73 (202).

Given the clinical relevance of tissue inflammation control in chronic Chagas disease, T_reg_ cells have been the subject of several lines of research. Thus, it has been determined that this lymphocyte subset is augmented in asymptomatic patients as compared to those with cardiac or digestive symptoms (199, 203–206). Furthermore, the existence of IL-10-producing activated T_reg_ cells has been demonstrated in both asymptomatic and cardiac chronic Chagas disease patients (207), while this cytokine shows higher serum concentration in asymptomatic patients than in cardiac ones (208). Also, it has been suggested that T_reg_ cells from severe and moderate cardiac Chagas patients have less suppressor capacity than those from asymptomatic and mild cardiac patients, and non-infected individuals (199). In a murine IL-10 deficient models of infection, an increased presence of CD8^+^ and CD4^+^ T cells in inflammatory cardiac infiltrates, albeit with the exception of T_reg_ cells, which have a decreased frequency (209). IL-10 knock-out infected mice have elevated levels of IL-12, TNF-α and IFN-γ in serum, which is associated to a reduced parasitaemia and parasite burden, but also suffer weight loss, hypothermia and have worse survival rate (196, 210). These conditions are ameliorated upon administration of exogenous IL-10 (210) or TNF-α blockade (196). In accordance, anti-GITR (211, 212) treatment of infected mice has similar effects in the composition of these infiltrates, and animals exhibit increased TNF-α production, increased heart parasitism and worse survival rate (213). The production of TGF-β, was discarded as the cause of the lack of cytokine production observed in effector CD8^+^ T cells isolated from inflammatory infiltrates in *T. cruzi* infected heart tissue, but it regulates the expansion of these cells and therefore has a protective role against cardiopathy (214, 215).

As for the specificity of CD4^+^ T cell response in human *T. cruzi* infection, exploration has been scarce, especially when compared to the extent to which CD8^+^ T cells specificity has been studied so far. Some chronic Chagas patients show CD4^+^ T cell response to TSA-1 and Tc24, two antigens that had been tested as vaccine candidates in preclinical animal models (216). KMP-11 contains CD4^+^ T cell-activating epitopes as well, as its been proven that it induces IFN-γ secretion and proliferation in certain *T. cruzi* infected subjects (188). However, it is important to notice that these antigens triggered a recall response in only a fraction of the patients within the cohorts of their respective studies, which might indicate that these proteins contain HLA-restricted epitopes.

## Concluding remarks

Across several decades of research, a remarkable volume of information regarding the immune response to *T. cruzi* infection and its role in protection, chronicity, inflammation and pathology has accumulated. Thanks to the development of different experimental models and the combined effort of researchers from diverse disciplines, many of the mechanisms and players underlying this host-pathogen interaction have been unveiled. The motivation behind this review was to put together the information so far collected in an organized, comprehensive manner, in the way of those who sort the pieces of a jigsaw puzzle on a table, following a particular criterion, before they set to assemble them into a sensible picture.

It is tempting to speculate that an immune-intervention strategy could help ameliorate (if not cure) chronically infected patients. Besides, and given the environmental factors that influence Chagas disease transmission, prophylactic vaccination can be envisaged as the ultimate tool for its prevention. Yet, these possibilities are critically hampered by our lack of understanding of the integral panorama of immunity in the context of *T. cruzi* infection. Success in translating this enormous amount of information into concrete applications has been scarce, and it's urgent. In the authors' view, the reason behind this lies in the complexity of the parasite-host interaction, a common factor to most protozoan infections which can hardly be comprised by the traditional, reductionist strategies. Most research in the field has focused on clarifying the role and relevance of individual cell subsets or activation profiles for controlling the infection, and their relationship with chronic Chagas disease pathology. But these approaches do not allow an integrative comprehension of anti-*T. cruzi* immunity, especially considering the high degree of interconnection and interdependence this system displays. In that sense, high throughput technologies and systems biology applied to immunology are tools that this field has just began to benefit from, and which are expected to bring new insights into this intricate interplay.

In conclusion, our knowledge of this matter has come a long way, but there is a clear need for the dots to be connected in order to drive science in the direction of making an actual difference for the millions of people affected by this neglected disease.

## Author contributions

GA, MG, and KG designed and implemented the review, performed literature search, and wrote the manuscript. All authors contributed to manuscript revision, read, and approved the submitted version. GA designed and produced illustrations.

### Conflict of interest statement

The authors declare that the research was conducted in the absence of any commercial or financial relationships that could be construed as a potential conflict of interest. The reviewer AP declared a shared affiliation, though no other collaboration, with the authors to the handling Editor.
