# Supplementary material for: The Unsolved Jigsaw Puzzle of the Immune Response in Chagas Disease
Source: Front Immunol. 2018 Aug 24;9:1929. doi: 10.3389/fimmu.2018.01929 (PMC6117404; doi:10.3389/fimmu.2018.01929)
Supplement: Supplementary file 1 [file Image_1.pdf]

Supplementary Material

The unsolved jigsaw puzzle of the immune response in Chagas disease

Gonzalo R. Acevedo, Magalí C. Girard, Karina A. Gómez*

*** Correspondence:** Corresponding Author: gomez@dna.uba.ar

1.1 Supplementary Figures

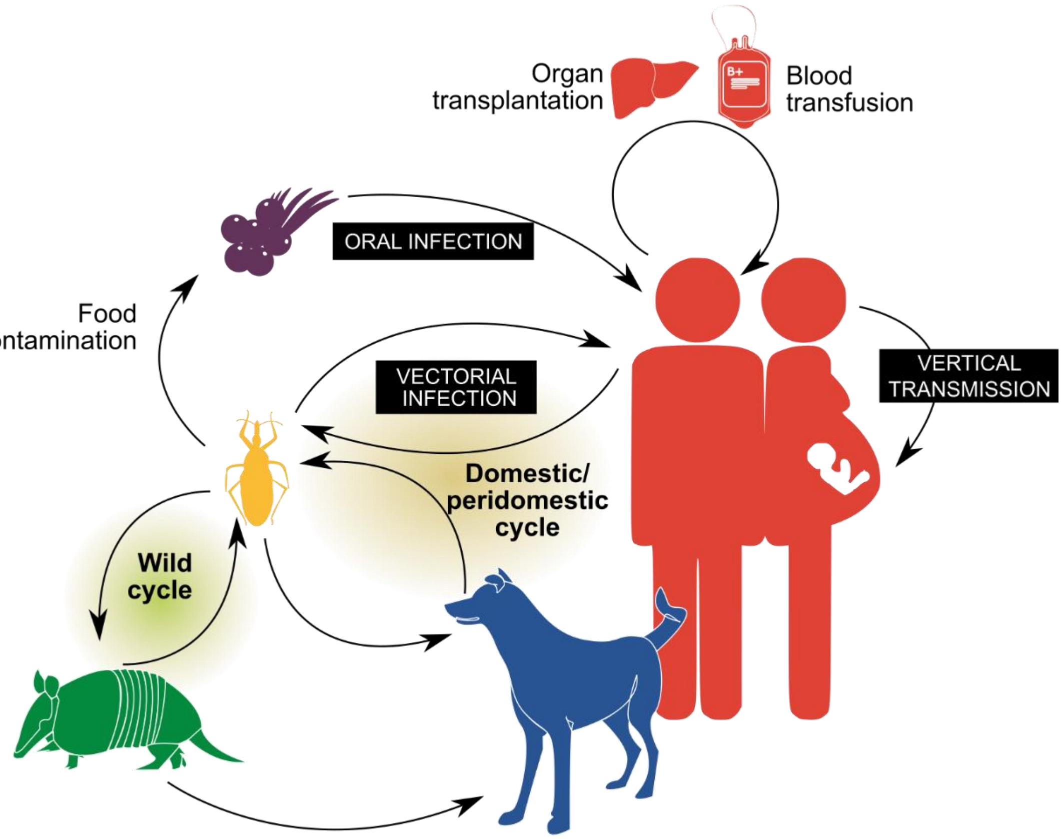

Supplementary Figure 1. Different routes and cycles of *T. cruzi* transmission.

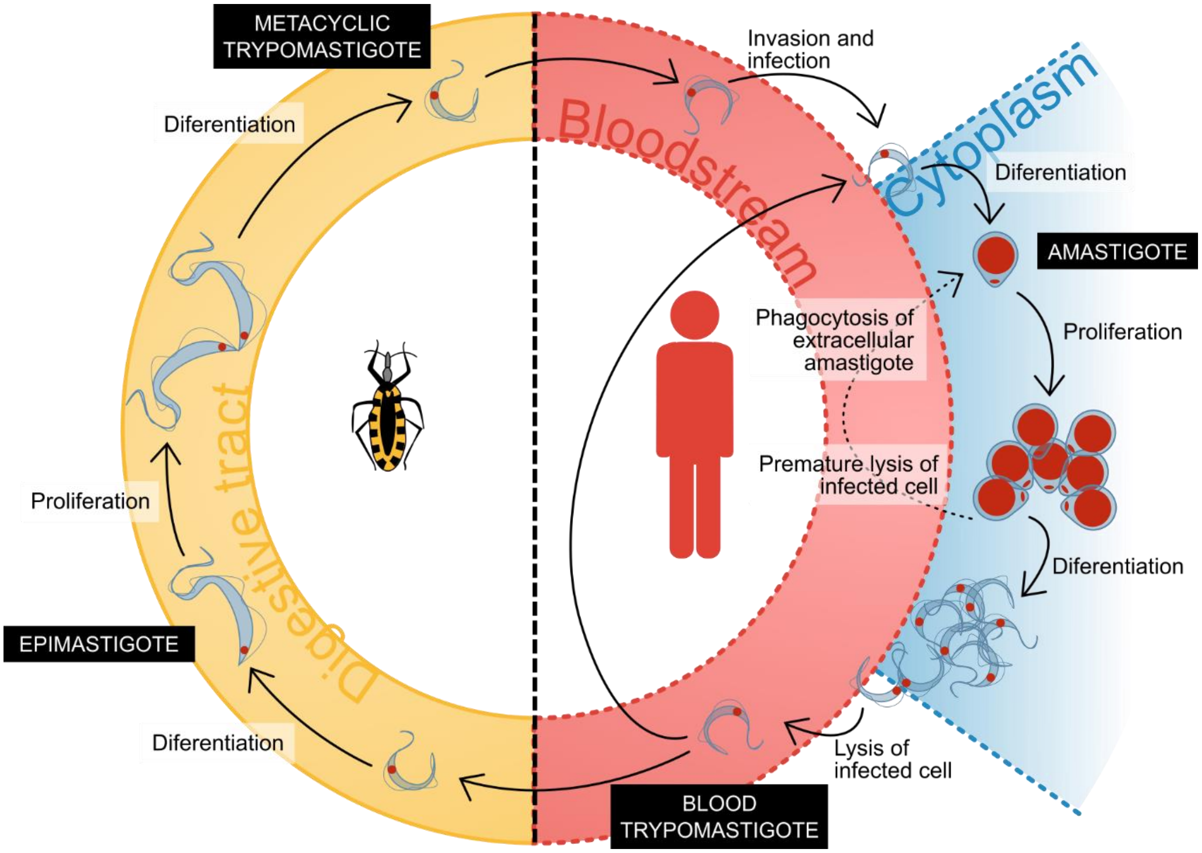

.

Supplementary Figure 2. *T. cruzi* life cycle

T. cruzi has a complex life cycle characterized by the presence of three main stages that can be easily identified by optical microscopy techniques, according to morphological criteria such as the presence of flagellum and relative position of the kinetoplast with respect to the nucleus. The parasite has two different hosts during its life cycle, a hematophagous vector insect belonging to the subfamily Triatominae (Hemiptera and Reduviidae) and a mammal host.

Trypomastigotes are ingested by the vector insect from an infected human (or another animal, wild or domestic) along with the blood. Once inside the digestive tract of the insect, they differentiate into epimastigotes and multiply by binary division, forming a reservoir of parasites in this host. Epimastigotes migrate down the tract and, in the rectum, they adhere to the cuticle and differentiate into metacyclic trypomastigotes, the non-replicative, infectious stage of the life cycle. These are released with the feces upon blood intake by the insect. Metacyclic trypomastigotes enter the human organism through mucous membranes or skin wounds. Once a trypomastigote penetrates a cell, it differentiates into the amastigote form, which is capable of proliferating by binary division and, subsequently, differentiate into blood trypomastigote. This form is released when the cell host breaks down, and may infect nearby cells, migrate through the bloodstream to invade distant tissues, or be ingested again by a vector, thereby closing the life cycle.

In addition, there is an alternative sub-cycle that is established when extracellular amastigotes, originated by the premature rupture of the host cell or by extracellular trypomastigote differentiation, are engulfed by macrophages, where they can survive and complete the intracellular cycle.
